# Competence-based performance evaluation in hospital nurses^*^

**DOI:** 10.1590/1518-8345.3173.3184

**Published:** 2019-10-14

**Authors:** Mirelle Inácio Soares, Laura Andrian Leal, Zélia Marilda Rodrigues Resck, Fábio de Souza Terra, Lucieli Dias Pedreschi Chaves, Silvia Helena Henriques

**Affiliations:** 1 Centro Universitário de Lavras, Unilavras, Lavras, MG, Brasil.; 2 Universidade de São Paulo, Escola de Enfermagem de Ribeirão Preto, Centro Colaborador da OPAS/OMS para o Desenvolvimento da Pesquisa em Enfermagem, Ribeirão Preto, SP, Brasil.; 3 Bolsista do Conselho Nacional de Desenvolvimento Científico e Tecnológico (CNPq), Brasil.; 4 Universidade Federal de Alfenas, Escola de Enfermagem, Alfenas, MG, Brasil.

**Keywords:** Nurses, Professional Competence, Hospitals, Employee Performance Appraisal, Strategies, Learning, Enfermeiros, Competência Profissional, Hospitais, Avaliação de Desempenho Profissional, Estratégias, Aprendizagem, Enfermeros, Competencia Profesional, Hospitales, Evaluación Del Rendimiento de Empleados, Estrategias, Aprendizaje

## Abstract

**Objective:**

to evaluate the frequencies attributed to the professional competences of hospital nurses, discussing the ones that obtained higher and lower frequencies.

**Method:**

descriptive, cross-sectional, quantitative study with 45 nurses of a hospital of high complexity in the interior of São Paulo state. The study used the Competences Evaluation Questionnaire, translated and validated in Brazil, composed of 27 items in five domains: professionalism, communication, management, nursing process and problem solving. Data were analyzed using descriptive statistics.

**Results:**

it was found that 80% of the participants were female and 20% were male. The age ranged between 25 and 63 years, with an average of 40.02 years, and the average length of professional experience was 13.39 years. The competences “Communication with the hospital’s administrative staff” and “Participation in scientific research and / or application of results” had the lowest frequencies, while “Commitment to punctuality and workload” and “Commitment to the ethical principles of the profession” were evaluated with the highest frequencies.

**Conclusion:**

evaluating the performance by competences becomes essential for managers and training centers, since it contributes to the identification of gaps in knowledge, skills and attitudes of professionals, by promoting the elaboration and implementation of strategies for their development.

## Abstract (pt)

**Objetivo:**

avaliar as frequências das competências profissionais de enfermeiros hospitalares, discutindo aquelas de maior e menor frequência atribuídas.

**Método:**

estudo descritivo, transversal e quantitativo, com 45 enfermeiros pertencentes a um hospital de alta complexidade do interior paulista. Utilizou-se o Questionário de Avaliação de Competências, traduzido e validado no Brasil, composto por 27 itens em cinco domínios: Profissionalismo, Comunicação, Gerenciamento, Processo de enfermagem e Resolução de problemas. Para a análise dos dados empregou-se a estatística descritiva.

**Resultados:**

constatou-se que 80% dos participantes correspondiam ao sexo feminino e 20% ao masculino. A idade variou entre 25 e 63 anos, com média de 40,02 anos e a média do tempo de experiência profissional foi de 13,39 anos. As competências “Comunicação com o pessoal administrativo do hospital” e “Participação em pesquisas científicas e/ou aplicação de resultados” tiveram as frequências menores; já o “Comprometimento com a pontualidade e a carga horária de trabalho” e o “Compromisso com as diretrizes éticas da profissão” foram avaliadas com as frequências mais elevadas.

**Conclusão:**

avaliar o desempenho por competências torna-se imprescindível para os gestores e os centros formadores, pois contribui para a identificação das lacunas de conhecimentos, habilidades e atitudes dos profissionais, promovendo a elaboração e a implementação de estratégias para o seu desenvolvimento.

## Abstract (es)

**Objetivo:**

evaluar las frecuencias de las competencias profesionales de enfermeros hospitalarios, discutiendo aquellas a las que se atribuyen mayor y menor frecuencia.

**Método:**

estudio descriptivo, transversal, cuantitativo, con 45 enfermeros pertenecientes a un hospital de alta complejidad del interior paulista. Se utilizó el Cuestionario de Evaluación de Competencias, traducido y validado en Brasil, compuesto por 27 ítems en cinco dominios: Profesionalismo, Comunicación, Gestión, Proceso de enfermería y Resolución de problemas. Para el análisis de los datos se empleó la estadística descriptiva.

**Resultados:**

se constató que un predominio del sexo femenino, con el 80 % de los participantes contra el 20% del sexo masculino. La franja etaria varió entre 25 y 63 años, con edad media de 40,02 años y el promedio del tiempo de experiencia profesional fue de 13,39 años. Las competencias «Comunicación con el personal administrativo del hospital» y «Participación en investigaciones científicas o aplicación de resultados» obtuvieron las menores frecuencias; mientras que el «Compromiso con la puntualidad y la carga horaria laboral» y el «Compromiso con las directrices éticas de la profesión» fueron evaluadas con las frecuencias más elevadas.

**Conclusión:**

evaluar el desempeño por competencias se hace imprescindible para LOS gestores y los centros formadores, ya que contribuye a la identificación de las brechas de conocimientos, habilidades y actitudes de los profesionales, con lo que promueve la elaboración e implementación de estrategias para su desarrollo.

## Introduction

The professional competences have been the object of concern and attention for the managers, and even for the nurses who work in the management and in the care. In this sense, identifying and developing them became a challenge and focus of interest for all actors involved, especially in the hospital setting^1^.

In this respect, the routine and systematic evaluation of the frequency of nurses’ competences has gained attention among educators, health managers and other professionals in different spheres, allowing to rescue the profile of competences, their trajectory and the achievement of objectives along the professional career^2^.

The nurse who works in the hospital service stands out for the technical responsibility in caring, both for his or her know-how and for the activities developed by the team, without neglecting the administrative part that this nurse may be responsible for. This multiplicity of actions requires the development of specific skills for professional practice at the level of excellence. In this perspective, managers should be concerned with the assessment of the professionals’ performance through instruments that evaluate their competences for the working praxis. Thus, having an instrument that identifies existing competences and those that need to be acquired becomes significant for distinguishing the singularity of actions for a professional practice which is safe, humane and with no risk to the client, the nurse or the institution^3^.

In view of this, the performance evaluation represents an essential tool that can characterize the profiles of professionals, that is, their cognitive, procedural and attitudinal knowledge, in this case especially of nurses and based on this diagnosis, to build and implement strategies to promote their learning. Thus, it is noteworthy to emphasize that this type of evaluation is increasingly disseminated in academic and organizational scenarios, given its capacity to provide management aligned with corporate strategic objectives and individual expectations^4^.

In this context, a study pointed out the need to build a specific instrument capable of verifying the professional competence of nurses working in emergencies, anchored in the profile of the professional, the client, the institution and the Brazilian public policy for the care in this area, according to a proper methodology^3^.

Moreover, it is important to highlight that the processes of characterization of professional profiles can begin even before the stage of hiring the workers, that is, during the selection and recruitment process carried out by the institution, since this movement also assists in the adequate allocation of workers, so that they effectively correspond to the position established. In this case, the personnel manager must establish a plan for the recruitment and permanent education processes, since misleading hiring has a negative impact on the performance of each function, directly reflecting on job satisfaction, on the professional engagement, as well as on the evaluation of results for the users and financial resources^5-6^.

Thus, in view of the complexity of hospital organizations, of the increasing demands of users of these services and for activities performed by nurses, this study assumes that some competences needed for the execution of these activities may be lacking for these professionals. In this context, the following questions are presented: Which competences are more frequently noticed by hospital nurses during their professional practice? What competences need to be developed?

Regarding the relevance of this subject, it is possible to notice that in the area of health, the daily professional practice of nurses has not been regularly evaluated, largely due to the management model implemented. In this perspective, this research can allow the identification of higher and lower frequency competences within the hospital by the nurses themselves, with the purpose of preparing a worker with innovative and creative performance, capable of a social and critical reflection/action, an individual who constructs knowledge for the professional practice of Nursing. He or she may also help in the construction of Pedagogical Political Projects whose central axis is the training of nurses by competences.

Thus, the present study aims to evaluate the frequencies attributed to the professional competences of hospital nurses, discussing the ones that obtained higher and lower frequencies.

## Method

It is a descriptive, cross-sectional study, using a quantitative approach. The study scenario was a public teaching hospital institution, reference in urgent and emergency care and located in the interior of São Paulo state. The research period was from November 2016 to March 2017. The study population consisted of 45 nurses working in five hospitalization units of the referred institution, among the following: Pediatric, Psychiatry, Medical Clinic, Surgical Clinic and Neurology.

The inclusion criterion required taking nurses from both positions: management and care; performing their duties for more than six months in the institution, assuming that this time is enough to identify the necessary competences for their work. Nurses who were on vacation or other kinds of leave for health reasons were excluded.

The study began with the application of a sociodemographic questionnaire presenting a script with data on professional training and performance, such as: sex, age, experience, level of education, *lato sensu* or *stricto sensu* post-graduation and time of service.

For the evaluation of competences, the Competences Evaluation Questionnaire (QAC)^1^ was translated, semantically and psychometrically validated during the year 2016 and culminated in the habilitation thesis by one of the authors of this research, in 2017. However, it is worth to mention that the article extracted from the thesis, that includes the psychometric validation of the instrument, was only recently published, in the year 2018, due to the time length of procedures for the journal publication.

The QAC is a five-point *Likert* scale, consisting of 27 competence items, distributed in five domains: professionalism, communication, management, nursing process and problem solving. In this instrument, respondents should manifest themselves on a scale of 1 to 5, where 1 = not applicable and 5 = excellent. The answers of the five-point scale would vary according to the example: (check an option) (1- Not applicable; 2- Low frequency; 3- Moderate frequency; 4- Good; 5- Excellent)^1^.

We should point out that both questionnaires were applied in the nurses’ hospitalization units, with an explanation about the instruments. In this context, the self-completion was done by the participant, with no possibility of communication between the researcher and the respondent.

Given this premise, the self-appraisal consists in the nurses evaluating their own attitudes/behaviors inherent to their daily care practices, according to the degree/level of competence attributed to each one^7^.

The results were tabulated in the Statistical Package for the Social Sciences (SPSS), version 20.0, for the analysis of descriptive statistics of all the variables raised in the sociodemographic instrument, as well as through the QAC.

The study was approved by the Research Ethics Committee of the University of São Paulo at Ribeirão Preto College of Nursing, CAAE 58559416.3.0000.5393. Participants signed the Free and Clarified Consent Term for research with human beings guaranteeing their anonymity.

## Results

In the studied population of 45 nurses, we found that 80% (n=36) of subjects were female and 20% (n = 9) were male. The age ranged between 25 and 63 years, with an average of 40.02 years, and the average length of professional experience was 13.39 years. In terms of training, 40% (n = 18) had only undergraduate studies, 42.2% (n = 19) had a *lato sensu* graduate degree and 17.7% (n = 8) had a *stricto sensu* graduate degree, being 13.3% (n = 6) masters and 4.4% (n = 2) doctorates. Regarding the area of expertise *lato sensu*, there was predominance of Infectology and Urgency and Emergency (6.7%, n=3 for each of these areas), followed by Nursing Management (4.4%, n = 2).

When asked about the preparation for the current activity, 42.2% (n = 19) reported that they were not trained and 57.8% (n=26) answered positively, being prepared in specific admission training of the working institution and trained by *lato sensu* and *stricto sensu* post-graduation courses.

In this direction, Tables 1 and 2 present the descriptive statistics of the collected data, allowing to describe the frequency (n) and percentage (%) of the competence items of the QAC according to the nurses’ responses distributed in their respective domains (n=45).

**Table 1:** Distribution of competences by the Professionalism and Communication domains perceived by nurses working in the hospital context (n=45). Ribeirão Preto, SP, Brazil, 2016-2017

Competences and Domains	Frequency(n)/Percentage (%)				

	1*	2^†^	3^ǂ^	4^§^	5^ǀǀ^
Professionalism					
1.Personal appearance and professional attitude	00 (00,0)	03 (06,7)	05 (11,1)	15 (33,3)	22 (48,9)
2. Commitment to punctuality and workload	00 (00,0)	00 (00,0)	02 (04,4)	09 (20,0)	34 (75,6)
7.Collect and enforce hospital rules and regulations	00 (00,0)	01 (02,2)	02 (04,4)	18 (40,0)	24 (53,3)
8. Commitment to the ethical guidelines of the profession	00 (00,0)	01 (02,2)	01 (02,2)	12 (26,7)	31 (68,9)
9. Conceptual knowledge of nursing (familiarity with the basic sciences and theoretical concepts of nursing)	00 (00,0)	01 (02,2)	05 (11,1)	15 (33,3)	24 (53,3)
Communication					
3.Communication with patients and their families	00 (00,0)	02 (04,4)	06 (13,3)	13 (28,9)	24 (53,3)
4.Communication with physicians and other members of the multidisciplinary team	00 (00,0)	03 (06,7)	03 (06,7)	12 (26,7)	27 (60,0)
6.Communication with fellow nurses	02 (04,4)	01 (02,2)	03 (06,7)	11 (24,4)	28 (62,2)
25.Maintaining patient safety	01 (02,2)	01 (02,2)	04 (08,9)	20 (44,4)	19 (42,2)
26.Documentation of nursing activities	01 (02,2)	02 (04,4)	08 (17,8)	21 (46,7)	13 (28,9)
27.Communication of the activities with the nursing team	00 (00,0)	03 (06,7)	02 (04,4)	23 (51,1)	17 (37,8)

*1 = Does not apply; ^†^2 = Low frequency; ^‡^3 = Moderate frequency; ^§^4 = Good; ^**ǀǀ**^5 = Great

**Table 2:** Distribution of competences by the domains Management, Nursing Process and Problem Solving perceived by nurses working in the hospital context (n=45). Ribeirão Preto, SP, Brazil, 2016-2017

Competences and Domains	Frequency(n)/Percentage (%)				

	1*	2^†^	3^ǂ^	4^§^	5^ǀǀ^
Management					
5.Communication with administrative staff of the hospital (human resources and finance sectors)	02 (04,4)	16 (35,6)	13 (28,9)	04 (08,9)	10 (22,2)
12.Participation in scientific research and/or application of results	06 (13,3)	12 (26,7)	09 (20,0)	10 (22,2)	08 (17,8)
21. Generation of new knowledge related to the development of the profession	01 (02,2)	08 (17,8)	10 (22,2)	14 (31,1)	12 (26,7)
22.Administrative and accountability skills	04 (08,9)	08 (17,8)	12 (26,7)	11 (24,4)	10 (22,2)
23. Enthusiasm and motivation in conducting nursing activities	04 (08,9)	07 (15,6)	13 (28,9)	09 (20,0)	12 (26,7)
24. Appropriate Application of hospital philosophy and procedures	02 (04,4)	04 (08,9)	16 (35,6)	15 (33,3)	08 (17,8)
Nursing Process					
10.Safety in the implementation of nursing skills	00 (00,0)	03 (06,7)	01 (02,2)	23 (51,1)	18 (40,0)
11.Updating of knowledge in the nursing area	00 (00,0)	05 (11,1)	07 (15,6)	17 (37,8)	16 (35,6)
13.Knowledge of the stages of the nursing process	00 (00,0)	04 (08,9)	04 (08,9)	23 (51,1)	14 (31,1)
14. Ability to carry out the steps of the nursing process	00 (00,0)	04 (08,9)	07 (15,6)	17 (37,8)	17 (37,8)
16.Capacity to perform accurate and precise nursing diagnoses	00 (00,0)	01 (02,2)	04 (08,9)	17 (37,8)	23 (51,1)
Problem solving					
15. Ability to assess the patient’s biological, psychological, social and spiritual needs	00 (00,0)	03 (06,7)	05 (11,1)	16 (35,6)	21 (46,7)
17. Ability to establish priorities in patient care	01 (02,2)	01 (02,2)	01 (02,2)	15 (33,3)	27 (60,0)
18.Execution of nursing responsibilities based on appropriate scientific foundation	00 (00,0)	03 (06,7)	05 (11,1)	22 (48,9)	15 (33,3)
19.Appropriate handling of critical patients	00 (00,0)	02 (04,4)	03 (06,7)	20 (44,4)	20 (44,4)
20.Efficient use of time at work	01 (02,2)	01 (02,2)	07 (15,6)	21 (46,7)	15 (33,3)

*1 = Does not apply; ^†^2 = Low frequency; ^‡^3 = Moderate frequency; ^§^4 = Good; ^||^5 = Great

According to Tables 1 and 2 and through descriptive statistics, the most and least frequent competences were highlighted in the routine of the nurses’ work process in the “Professionalism”, “Communication” and “Management” Domains.

## Discussion

Through the results of the competence frequencies, we observed that the nurse becomes competent in what concerns the commitment with punctuality and respect to the workload.

It is common knowledge that a hospital organization is considered effective and efficient when it presents excellence in quality and a good performance of its human capital^8^. In view of this, when nurses have an ethical attitude and responsibility to commit themselves to work and to users, this makes them an empowered professional able to make decisions in the most varied situations of daily work.

Thus, being assiduous and respecting the workload, is how nurses, through the collective construction, achieve forms of leadership, relationship, communication and action, strengthening both the professional and the user of health services, through the valorization of the reciprocal relationship between work and interaction^9^.

In association with the issue of punctuality and workload, commitment to the ethical guidelines of the profession and of the skills in setting priorities in patient care have also brought the relevance in which nurses are willing to work within the ethical and legal context of the profession.

In this sense, a good nurse is built for his/her visibility in society, through an appropriate and respectful posture, since what promotes the qualification of nursing work is not only knowledge, but also the way of acting, the professional stance which are, directly, reflected in the effectiveness of care^10^.

Another competence that occurs frequently is the communication with doctors and other members of the multidisciplinary team and the communication with nurse fellows, realizing that there is concern in interacting with all the team that provides assistance to the user. This effective contact is one of the most relevant points to be absorbed, since the result of a favorable communication makes it possible to standardize actions, share knowledge and values, and facilitate relations with the internal and external public^11^.

Effective internal communication promotes the development and maintenance of relationships between institution, managers and employees, by sharing ideas, experiences and knowledge, contributing to a greater involvement of professionals in the work environment, while facilitating employees ability to link their values and objectives to those of the organization^12^.

Thus, communication in any health institution should be understood as an essential competence for professionals, also serving as a tool in the search for success and for obtaining organizational results, since in a hospital people perform functions of extreme importance, so it is essential for them to be well informed, so that they can become involved and participate in managerial actions, helping the accomplishment of predetermined goals and objectives^13^.

Regarding the relevance of effective communication at work, it was possible to identify that some forms of communication present low frequency, that is, not much identified or performed by nurses. It is the case of “communication with the hospital’s administrative staff”. Although nurses emphasize effective communication with the multidisciplinary team and among their colleagues, this fact appears limited when it is about the administrative staff of the institution.

This may be related to the fact that nurses, especially those in care positions, have to go through several hierarchical levels of the hospital administration to inform any type of problem situation in the unit. Thus, they should refer to their direct superiors who, in turn, go to their respective supervisors and only then the information will go to higher instances of the hospital.

It is believed that the flow of information transmission through several hierarchical levels and through several professionals can cause ineffective or disturbed communication; therefore, it can cause damages in the services provided by these professionals. In this sense, we should say that the organizational structure of the institution directly affects the process of communication between its constituents.

It is worth to mention that communication is one of the five main problems that affects patient safety; so actions aimed at improving the process of issuing and transmitting information are part of the global guidelines for standardization and implementation of protocols for the reduction of adverse events , greater security and quality of care^14^.

It is known that weaknesses in communication can have their origin in the lack of teamwork, lack of training of professionals, the non-use of standardized instruments, as well as interpersonal conflicts in the institution^15^.

It is also known that in places where communication is ineffective among professionals, the transmission of information may occur incompletely and due to this, will interfere in the access and participation in informative or educational activities happening inside the hospital, including research of a scientific nature carried out in partnership between the hospital organization and the training centers.

In this sense, the strategy of meetings allows reflection on the importance of relational abilities in nurses’ performance, especially in relation to observation, listening and communication. Therefore, weekly meetings between multiprofessional teams, giving proper *feedback* information and the involvement of health care professionals in the analysis of organization’s indicators are considered essential tools for the achievement of institutional goals, greater engagement and autonomy of the team^16^. The promotion of spaces for professionals to express their feelings and expose the conflicts that are happening in their work process is extremely relevant^17^.

Moreover, in addition to strategies to stimulate effective communication, the technological development and the increasing use of Information and Communication Technologies (ICT), applied to the educational and professional contexts, stimulate the adoption of devices that promote interaction between individuals, and this has also happened in the hospital scenario. The intention is to enable new communication resources, including *WhatsApp* communication application, which allows the exchange of text messages, images, sounds and videos, and is widely used in the social context^18^.

It is important to emphasize that the appropriation of technological resources allows nursing to take time in its activities, optimizing the work process. Thus, the nurse must be aware of the development of knowledge, skills and attitudes for the use of computational technologies^19^. The opportunity to identify and discuss ICT devices could enable an improvement in the competence of communication in multiprofessional meetings.

Therefore, the use of technology allows a more dynamic teaching-learning process of health professionals and users, offering a new form of teaching, more individualized, but also collective and participative, that respects the learning pace of each person, with potential to assist in a more humanized and higher-quality training and assistance^20^.

It is fundamental that managers and coordinators identify the needs of information technologies and the degree of knowledge of their peers in the institution, aiming at standardizing the language in the various communication channels to be used by those involved in the process.

Thus, the issue of concern over the theme of nursing research priorities is not new, but the continuity of its discussion is justified, at a time when the need to redefine areas of knowledge and the research topics that guide the scientific production in this field of knowledge are included in the agenda of the profession^21^.

In view of this, participating in research activities, scientific projects with other institutions, can be one of the ways to improve professional performance, since this research portrayed the low frequency of the involvement of nurses in scientific research.

Corroborating this fact, it has been observed that nurses tend to seek only specialization courses, since masters and doctoral degrees, although allowing some conditions for the quality of care, are associated with projects to build a teaching career^22^. And this premise also does not seem to cause any impact on the organization.

In this context, scientific research in nursing is considered relevant and essential, and its productivity is an icon of a capitalist way of thinking about the construction of knowledge^23^. Thus, studying, analyzing and reflecting critically on one’s own work, to identify strategies that can collaborate for the participation of nurses in scientific research, becomes a challenge.

Thus, participating in research is also to become part of research groups in the training institutions, as well as joining *lato sensu* or *stricto sensu* post-graduation programs and the trainings, which can be part of the organization’s permanent education strategies, aiming at the development of competences transforming the working praxis.

After the implementation of research in the practice, an environment of construction and socialization of knowledge can be created, aiming at a professional and assistance qualification. However, to make research develop as a consistent practice within health organizations, it is necessary, after recognizing the difficulties, to present effective planning and implementation alternatives^24^.

Thus, health institutions, being a school hospital in the case of this study, have a social responsibility toward their collaborators, in stimulating and developing investigations, not only for the benefit of internal, documentary issues, directed to the professionals, but especially for the quality of care to the user. In this direction, nursing managers must rethink whether the working praxis of nurses is scientifically grounded, as well as whether the professionals recognize the applicability of research in the context of practice.

The limits of the study were related to the fact that the research was carried out in a public teaching hospital institution, although it is a reference in emergency care, as well as the quantitative limitation of the number of participants of 45 nurses, making it difficult to provide a more consistent statistical analysis, including variables associations/correlations.

As a contribution to the advancement of scientific knowledge, the study emphasizes the benefit of using the performance evaluation tool focusing on competences of nurses in hospital institutions, so as to improve and strengthen knowledge, skills and attitudes of these professionals, as well as to broaden managers’ view of the reality of their institution. Consequently, this type of tool may lead to the adherence to Training and Development (T&D) plans of workers’ competences and career.

## Conclusion

The study allowed to evaluate the frequencies of hospital nurses’ competences, allowing the application of learning-teaching strategies to be developed in the work context.

Regarding the lower frequency competences, we observed that the effectiveness of communication with the administration and the participation in scientific research are essential in a hospital institution where the priority is the urgent and emergency care, since the information which continually passed to all hierarchical levels enable the continuity of actions and encourage the change of behavior and attitude of employees, when they understand the relevance of this process for the nurse’s working praxis.

In the sequence, the training of the nursing professional becomes relevant, so that he/she understands the importance of his/her participation and research achievement, as well as the application of the results in the daily work. However, the strategies of training and qualifications in the institution studied should be designed in order to meet the entire team in their various work shifts. It is known that access to information must be free through all hierarchical levels and for all professionals, especially the information related to their improvement.

In short, it was possible to verify the relevance of the application of instruments that characterize the profile of professionals, identifying gaps in knowledge, skills and attitudes that may be developed and improved through teaching-learning methodologies with the worker, aiming at the effective professional performance with positive repercussions on the care provided.

## References

[1] Henriques SH, Soares MI, Leal LA (2018). Applicability assessment of the portuguese version of a
competency questionnaire for hospital nurses. Texto Contexto Enferm.

[2] Finnbakk E, Wangensteen S, Skovdahl K, Fagerstrom L (2015). The professional nurse self-assessment scale: psychometric
testing in Norwegian long term and home care context. BMC Nurs.

[3] Holanda FL, Marra CC, Cunha ICKO (2018). Assessment of professional competence of nurses in emergencies:
created and validated instrument. Rev Bras Enferm.

[4] Jorge FC, Bittencourt JP, Galleli B (2014). Evaluation of competencies in a hospital institution: the view of
evaluators and evaluators. Future Stud Res J.

[5] Schuettner KA, Van Sell SL, Sheriff S (2015). Nursing administration degree as the foundation of practice for
future nurse managers. Nurse Leader.

[6] Watkins A, Wagner J, Martin C, Grant B, Maule K, Resh K (2014). Nurse manager residency program: an innovative leadership
succession plan. Dimens Crit Care Nurs.

[7] Holanda FL, Marra CC, Cunha ICKO (2019). Evidence of validity of the Competence Scale of Actions of Nurses
in Emergencies. Rev. Latino-Am. Enfermagem.

[8] Ariyani I, Haerani S, Maupa H, Taba MI (2016). The influence of organizational culture, work motivation and
working climate on the performance of nurses through job satisfaction,
organizational commitment and organizational citizenship behavior in the
private hospitals in Jakarta, Indonesia. Scientific Res J.

[9] Fernandes HN, Thofehrn MB, Porto AR, Amestoy SC, Jacondino MB, Soares MR (2015). Interpersonal relationships in work of multiprofessional team of
family health unit. J Res.: Fundam Care Online.

[10] Leal LA, Camelo SHH, Soares MI, Santos FC, Correa R, Chaves LDP (2016). Professional competences for nurses: the view of undergraduate
nursing students. Rev Baiana Enferm.

[11] Accreditation Canada (2015). The value and impact of health care accreditation: a literature
review.

[12] Karanges E, Johnston K, Beatson A, Lings I (2015). The influence of internal communication onemployee engagement: a
pilot study. Public Relations Review.

[13] Macêdo DF, Romeiro TIC, Marsiglia DC (2015). The importance of the administrator in hospital management:
perception of doctors, nurses and administrators of a university
hospital. Rev FOCO.

[14] World Health Organization (2014). The high 5s project: interim report.

[15] Lee JY (2015). Effective communication for patient safety. J Korean Med Assoc.

[16] Siman AG, Cunha SGS, Martins ES, Brito MJM (2015). Management strategies for hospital accreditation. REME Rev Min Enferm.

[17] Lima SBS, Rabenschlag LA, Tonini TFF (2014). Conflict management and resolution strategies for nurse
managers. Rev Enferm UFSM.

[18] Araújo PC, Bottentuit JB (2015). The WhatsApp communication application as strategy in teaching
Philosophy. Temática.

[19] Cardoso RB, Ferreira BJ, Martins WA, Paludeto SB (2017). Permanent education for the use of the electronic patient health
record in nursing. J Health Inform.

[20] Fonseca LMM, Tsai ML, Dias DMV, Scochi CGS, Fernandes AM, Martins JCA (2015). Emotional design and its contributions to digital educational
technology in health and nursing: integrative review. Referência.

[21] Oliveira DA, Gonçalves RS, Barbosa ACQ (2014). Human resources managers’ perception towards skills
management. Rev FSA.

[22] Melo WS, Oliveira PJF, Monteiro FPM, Santos FCA, Silva MJN, Calderon CJ (2017). Guide of attributes of the nurse’s political competence: a
methodological study. Rev Bras Enferm.

[23] Lino MM, Backes VMS, Costa MASMC, Martins MMFPS, Lino MM (2017). The influence of capitalism on the production of knowledge in
nursing. Rev Gaúcha Enferm.

[24] Puggina CC, Amestoy SC, Fernandes HN, Carvalho LA, Báo ACP, Alves FO (2015). Permanent education in healthcare: instrument of transformation
of nurses’ work. Rev Espaço para a Saúde.

